# Systematics of Australian Thrasorinae (Hymenoptera, Cynipoidea, Figitidae) with descriptions of Mikeiinae, new subfamily, two new genera, and three new species

**DOI:** 10.3897/zookeys.108.829

**Published:** 2011-06-17

**Authors:** J. Paretas-Martínez, C. Restrepo-Ortiz, M. Buffington, J. Pujade-Villar

**Affiliations:** 1 University of Barcelona. Faculty of Biology. Department of Animal Biology. Avda. Diagonal 645 - 08028 - Barcelona. Spain; 2 Systematic Entomology Laboratory, USDA, c/o NMNH, Smithsonian Institution, 10th & Constitution Ave NW. PO Box 37012 MRC-168, Washington DC 20013, USA

**Keywords:** Australia, Figitidae, Mikeiinae, *Cicatrix*, *Mikeius*, *Palmiriella*, *Thrasorus*

## Abstract

The Australian Thrasorinae are revised and *Mikeius* is transferred to Mikeiinae Paretas-Martínez & Pujade-Villar, **subfam. n.**, and *Mikeius clavatus* Pujade-Villar & Restrepo-Ortiz, **sp. n.**, is described. Two new genera of Thrasorinae are erected: *Cicatrix* Paretas-Martínez, **gen. n.,** including *Cicatrix pilosiscutum*(Girault), **comb. n.** from *Amblynotus*, *Cicatrix schauffi* (Buffington), **comb. n.** from *Mikeius***,** and *Cicatrix neumannoides* Paretas-Martínez & Restrepo-Ortiz, **sp. n.**; and *Palmiriella* Pujade-Villar & Paretas-Martínez, **gen. n.**, including *Palmiriella neumanni* (Buffington), **comb. n.** from *Mikeius*, *Thrasorus rieki* Paretas-Martínez & Pujade-Villar, **sp. n.**, is also described. A phylogenetic analysis of 176 morphological and biological characters, including all these new taxa and all genera previously included in Thrasorinae, was conducted. All subfamilies were recovered as monophyletic, with the following relationships: Parnipinae (Euceroptrinae (Mikeiinae (Plectocynipinae (Thrasorinae)))). A worldwide key to the subfamilies of Figitidae is provided that includes the new subfamily, as well as a key to genera Thrasorinae.

## Introduction

Figitidae (Hymenoptera: Cynipoidea) are parasitoids of the larvae of other insects, principally cyclorraphous Diptera (Ronquist 1999; Buffington et al. 2007). Ronquist (1999) seperated the figitids into nine subfamilies: Anacharitinae, Aspicerinae, Charipinae, Emargininae, Eucoilinae, Figitinae, Parnipinae, Pycnostigminae, and Thrasorinae; Parnipinae was referred to in the study but formally described later by Ronquist and Nieves-Aldrey (2001). Two new figitid subfamilies, Plectocynipinae (Ros-Farré and Pujade-Villar 2007) and Euceroptrinae (Buffington and Liljeblad 2008), have been erected recently to include genera previously included in Thrasorinae.

Thrasorinae is a stem group of figitids (Buffington et al. 2007) associated with galls of other wasps (Cynipoidea and Chalcidoidea) on various trees and bushes. They are parasitoids of the gall inducers or other hymenopteran inhabitants in the galls with which they are associated (Ronquist 1999; Buffington and Liljeblad 2008). Hence, the group is important for elucidating the evolutionary history of Figitidae, in particular, and the Cynipoidea as a whole, with its different life strategies of entomophagy and phytophagy. Prior to this study, Thrasorinae included the four genera *Thrasorus* Weld (two species: Australia), *Mikeius* Buffington (six species: Australia), *Myrtopsen* Rübsaamen (eleven species: two Holarctic and nine Nearctic), and *Scutimica* Ros-Farré (two species: Neotropical). Thrasorinae are characterized by the circumtorular impression (Fig. 2A, D, 3A), not present in any other figitids (Pujade et al. 2008; Ros-Farre and Pujade-Villar 2007; Ros-Farre and Pujade-Villar 2009; present study).

Following the examination of many undetermined specimens of Thrasorinae in the Australian National Insect Collection (ANIC) and the Queensland Museum (QM), as well as the type material of all species included in *Mikeius* Buffington, new questions arose regarding the taxonomy of Thrasorinae. First, an undescribed species of *Mikeius* was discovered (described herein); second, two species originally described in *Mikeius* were determined to render the genus polyphyletic, and new generic assignments are required; and third, phylogenetic analyses determined that the inclusion of *Mikeius* within Thrasorinae renders the subfamily paraphyletic with respect to Plectocynipinae. In response to these discoveries, Mikeiinae is described as a new subfamily to accommodate *Mikeius*, and species previously described in *Mikeius* are moved into other genera. In two cases, no current genus concept could accommodate these species, and the two new genera *Cicatrix*, gen. n., and *Palmiriella*, gen. n., are herein described. The goal of this study is to bring clarity to the taxonomic and phylogenetic relationships of these unusual groups of figitid wasps.

## Material and methods

List of Repositories

QMQueensland Museum, Brisbane, Australia (C. Burwell).

ANICAustralian National Insect Collection, CSIRO, Canberra, Australia (J. LaSalle).

*Specimen illustration and observation.* Environmental scanning electron micrographs (ESEM) were obtained at Barcelona University with the FEI Quanta 200 ESEM without any coating at 15 KV. Additional ESEM images were obtained either with a Hitachi TM3000 E-SEM, or an Amray 1810 SEM under a vacuum, using a lanthanum hexaboride electron source (LaB6) at 10 Kv, both housed at the National Museum of Natural History, Smithsonian Institution. Images were edited using Adobe CS4 Software (Adobe, Inc). The terminology for morphological structures comes from Richards (1977), Ronquist and Nordlander (1989), Ronquist (1995), Ros-Farré et al. (2000), and Ros-Farré and Pujade-Villar (2007), and the sculpture terminology follows Harris (1979). Measurements and abbreviations in the descriptions include: F1-F12, first and following flagellomeres; T3-T4, third and fourth abdominal tergites; antennal formula is given with the length:width ratio of each segment.

*Phylogenetic analysis.* Twenty-two taxa were included in the phylogenetic analysis (Table 1), representing all genera previously and currently included in Thrasorinae, and all new taxa and combinations described in this work. Three species of each genus were included (except for monotypic genera or those with less than three species), so as to capture the morphological diversity of each genus. *Parnips nigripes* (Barbotin, 1964) was chosen as an out-group based on Buffington et al. (2007). The analysis was based on a morphological dataset of 172 morphological and 4 biological characters modified from Buffington et al. (2007); the character list can be found in Appendix 1. These characters represent the variability in the external morphological diversity of all the species studied, excluding those characters present in only one species; characters utilized in previous phylogenetic studies are indicated. Due to their rarity, some species were not dissected and examined internally; characters requiring dissection for coding were left as ‘?’. The resulting data matrix (Appendix 2), which included 79 parsimony-informative characters, was analyzed using PAUP* (Swofford, 2002) employeing 10,000 multiple random addition sequences, followed by TBR swapping with branches of maximum length zero collapsed and steepest descent set to ‘off’. For bootstrap analyses (Felsenstein 1985), we employed a simple addition sequence with *Parnips nigripes* as the reference taxon, followed by 1000 bootstrap replicates, each replicate employing 100 TBR swapping replications.

**Table 1.** Taxa included in the phylogenetic analysis. OG: outgroup.

Higher taxon	Species
Parnipinae (OG)	*Parnips nigripes* (Barbotin, 1964)
Euceroptrinae	*Euceroptres primus* Ashmead, 1896
	*Euceroptres whartoni* Buffington & Liljeblad, 2008
	*Euceroptres montanus* Weld, 1926
Thrasorinae	*Scutimica flava* Ros-Farré & Pujade-Villar, 2007
	*Scutimica transcarinata* Ros-Farré & Pujade-Villar, 2007
	*Myrtopsen platensis* Diaz, 1975
	*Myrtopsen luederwaldti* Dettmer, 1928
	*Myrtopsen mimosae* Weld, 1926
	*Palmiriella neumanni* (Buffington, 2008)
	*Thrasorus pilosus* Weld, 1944
	*Thrasorus schmidtae* Buffington, 2008
	*Thrasorus rieki* sp. n.
	*Cicatrix pilosiscutum* (Girault, 1929)
	*Cicatrix schauffi* (Girault, 1929)
	*Cicatrix neumannoides* sp. n.
Plectocynipinae	N. gen., n. sp. *plectocynipine*
	*Plectocynips pilosus* Díaz, 1976
Mikeiinae, subfam. n.	*Mikeius hartigi* (Girault, 1930)
	*Mikeius grandawi* Buffington, 2008
	*Mikeius clavatus* sp. n.
	*Mikeius berryi* Buffington, 2008

## Descriptions

### Mikeiinae

Paretas-Martínez & Pujade-Villar subfam. n.

urn:lsid:zoobank.org:act:9A0F4DEB-C4CE-44E2-BAAC-D86A88DC25CE

http://species-id.net/wiki/Mikeiinae

Fig. 1

#### Type genus:

*Mikeius* Buffington, 2008.

#### Diagnosis.

**Figure 1.**
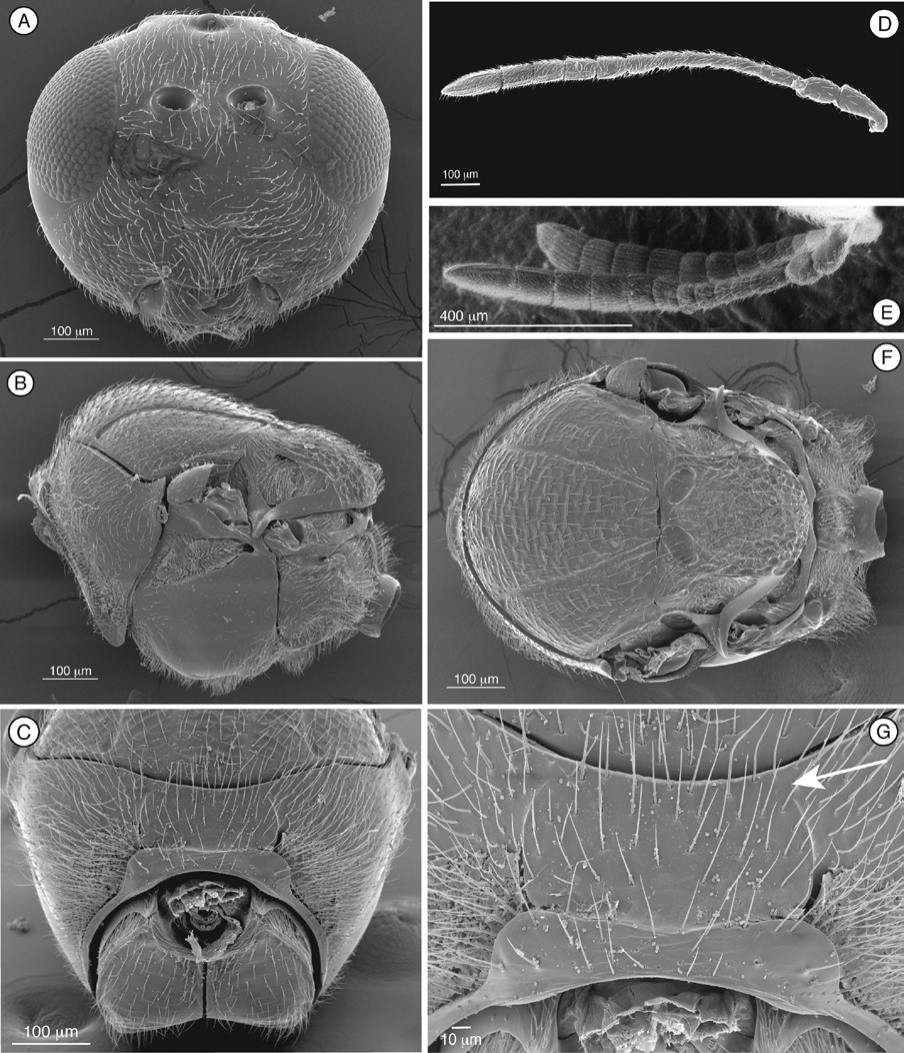
Diagnostic characters of *Mikeius* sp. (Mikeiinae), female. **A–D**, F and G: *Mikeius hartigi*; E, *Mikeius clavatus* **A** head, anterior view **B** mesosoma, lateral view **C** mesosoma, antero-dorsal view **D–E** antenna, medial view **F** mesosoma, dorsal view **G** pronotum (mesosoma), antero-dorsal view.

**Figure 2.**
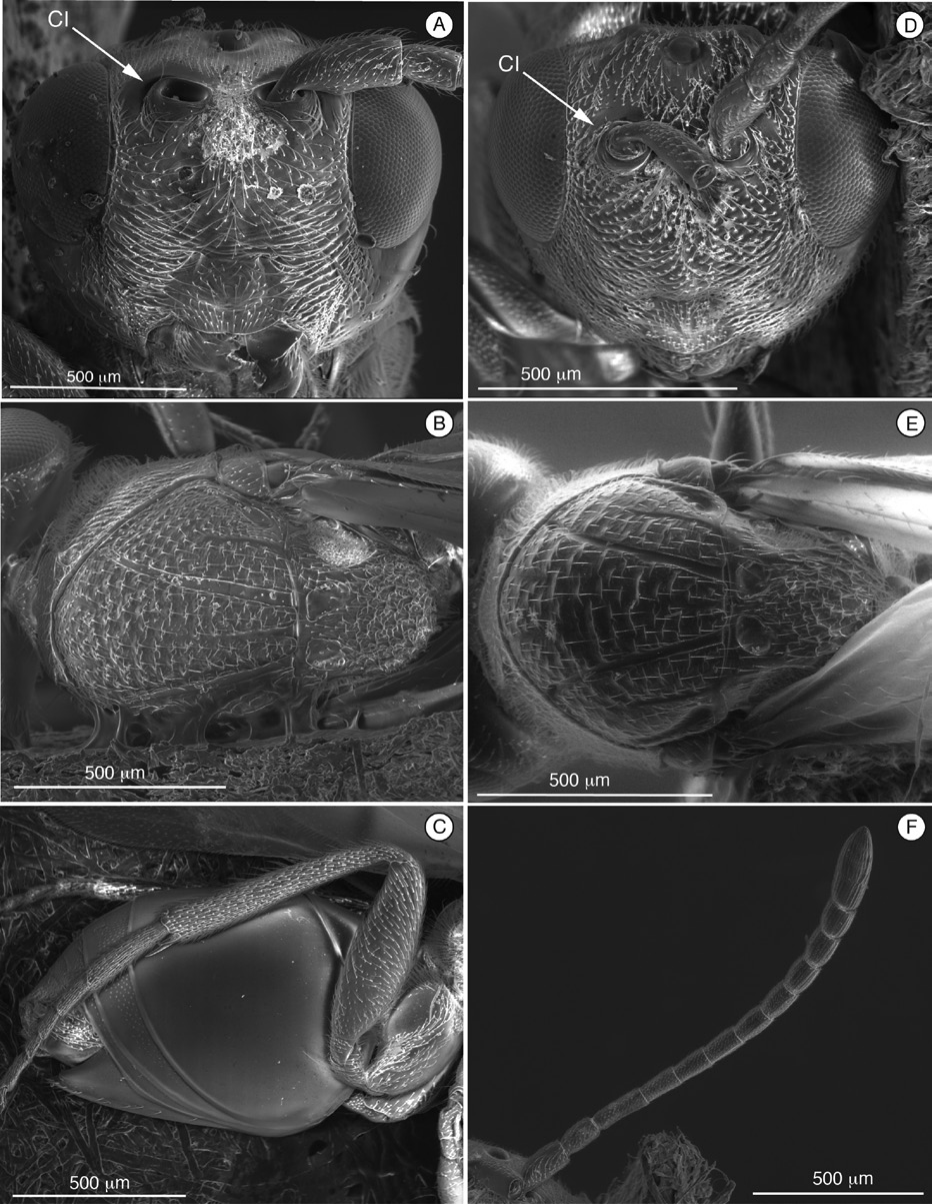
Diagnostic characters of *Cicatrix* sp. (Thrasorinae): **A** *Cicatrix pilosiscutum*; **B–D** and **F** *Cicatrix schauffi*; E, *Cicatrix neumannoides* **A** head, anterior view **B** mesosoma, dorsal view **C** metasoma, lateral view **D** head, anterior view **E** mesosoma, dorsal view **F**female antenna, dorsal view. CI, circumtorular impression.

**Figure 3.**
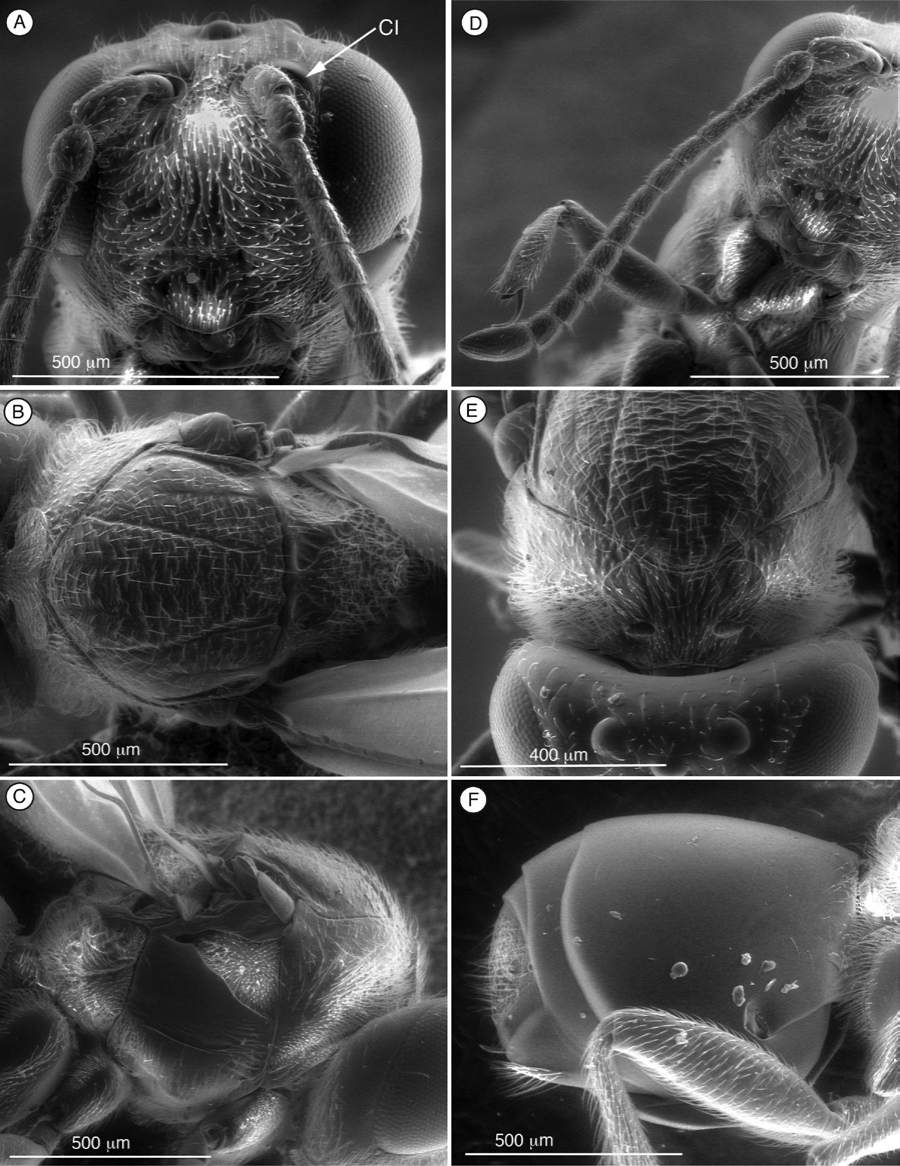
Diagnostic characters of *Palmiriella neumanni* (Thrasorinae), female **A**head, anterior view **B** mesosoma, dorsal view **C** mesosoma, lateral view **D** antenna, dorsal view **E** head and mesosoma, antero-dorsal view **F** metasoma, lateral view. CI, circumtorular impression.

**Figure 4.**
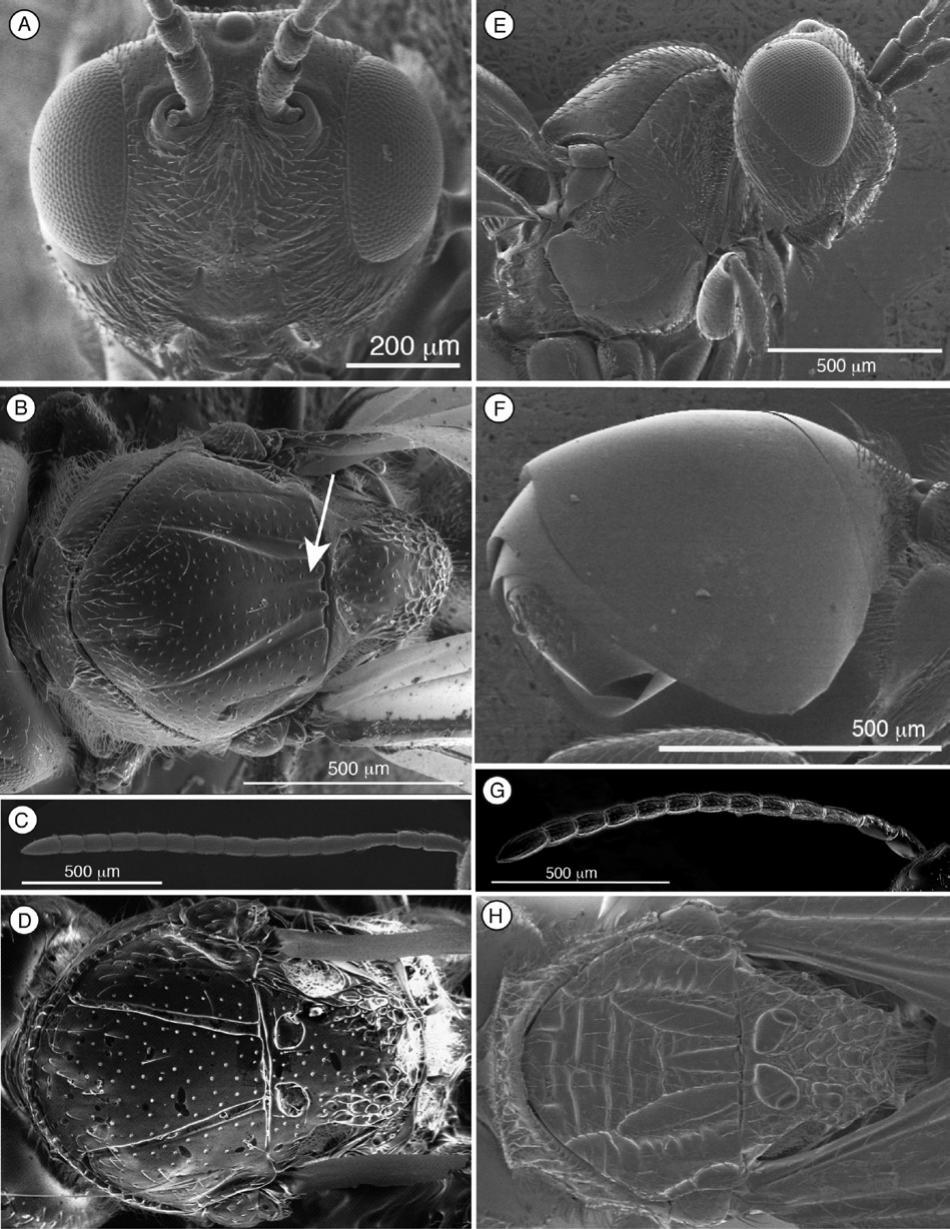
Characters of *Thrasorus* (**A–G**) and *Scutimica* (H) **A** head, anterior view **B** mesosoma, dorsal view, *Thrasorus rieki* **C** antenna, male, medial view **D** mesosoma, dorsal view, *Thrasorus schmidtae* **E** head and mesosoma, lateral view **F** metasoma, lateral view **G** antenna, female, medial view **H** mesosoma, dorsal view, *Scutimica transcarinata.*

Differs from Thrasorinae by the absence of a circumtorular impression (Fig. 1A; compare with Figs 2A, D, 3A, 4A, 9A-B), and the absence of a distinctly projected pronotal plate (Fig. 1C and G) (Table 2). Differs from Plectocynipinae by lacking an extremely long posterior metatibial spur (Fig. 6F; Ros-Farre and Pujade-Villar 2007), a laterally compressed metasoma in females (Ros-Farre and Pujade-Villar 2007), and a long, exposed hypopygium (7th sternite) in females (Ros-Farre and Pujade-Villar 2007). Differs from Euceroptrinae by lacking an areolet in the forewing, a lateral pronotal carinae (ARE, Fig. 6D; Buffington and Liljeblad 2008) and pronotal plate, having a complete ring of setae at the base of the metasoma and metasomal T4 much larger than T3. The Mikeiinae are unique among these three subfamilies in having two carinae in the median area of the pronotum that do not form a projected pronotal plate (Fig. 1G).

#### Description.

**Length**. 2 – 3.5 mm.

**Coloration.** Head and mesosoma dark brown to black, antenna and legs yellowish to brown. Metasoma light brown to black.

**Head.** (Fig. 1A) Frons and face with abundant setae. Transverse carinae or strigae on face absent. Clypeus distinctly projected ventrally, curved ventrally, clypeopleurostomal lines well developed. Malar furrow absent; malar space coriaceous, striate. Occiput and genae smooth without carinae. Circumtorular impression absent.

**Antenna.** (Fig. 1D, E) Filiform or clavate with 10–11 flagellomeres in females (last one larger, possibly fusion of two), 12 in males. Males with F1 curved.

**Mesosoma.** (Fig. 1B, C, F, G) Lateral margins of posterior part of pronotal plate short, not reaching scutum, not forming projected plate; lateral pronotal depressions open laterally. Mesoscutum horizontally striate. Notauli complete, uniformly wide along entire length, or gently widening posteriorly. Parascutal sulcus marked only in basal half. Lateral basal impressions weak. Antero-admedian lines absent or weak. Median mesoscutal line present, short or long. Scutellum striate anteriorly and in center, rugose posteriorly; scutellar foveae round subtriangular or subquadrate, sometimes not delimited posteriorly; interfoveal carina absent. Mesopleural furrow absent or present. Propodeal carinae wide, almost straight. Pronotum, mesoscutum, scutellum, mesopleural triangle and metapleura all covered with sparse/dense setae.

**Forewing.** Short setae present on wing surface and along margins. Radial cell closed along anterior margin, 2 to 2.5 times longer than wide, R2 almost straight; areolet absent.

**Legs**. Metatibia with two spurs, sub-equal in length, not exceeding one-third the length of tarsomere 1.

**Metasoma**. Base of T3 with a complete or incomplete ring of setae. Tergite 3 smaller than T4; T4 large, covering almost entire metasomal surface; remaining terga short, telescoped within T4; entire metasoma shiny and smooth.

#### Comments.

In the original description of *Mikeius*, Buffington (2008) erroneously described species of the genus as having 12 flagellomeres in the female antenna; the correct number is 10 or 11 (Fig. 1 D and E).

#### Biology.

Associated with Chalcidoidea (Hymenoptera: Apocrita) that induce galls on species of *Acacia* (Fabaceae)and *Eucalyptus* (Myrtaceae), although most of these host records await verification through isolated rearing (Buffington, 2008).

#### Distribution.

Australia.

#### Included genus.

*Mikeius* Buffington, 2008.

### Mikeius clavatus

Pujade-Villar & Restrepo-Ortiz sp. n.

urn:lsid:zoobank.org:act:8D74319A-2A25-48A6-B857-80E6ABB2BE9C

http://species-id.net/wiki/Mikeius_clavatus

Fig. 1E

#### Diagnosis.

Differs from all the other species of *Mikeius* in having the antenna strongly clavate with the six terminal segments 1.5 times wider than previous segments (Fig. 1E); further distinguished from *Mikeius berryi* and *Mikeius grandawi* by the absence of a mesopleural carina.

#### Description.

As in subfamily description (see above) with the following specific characters.

**Length**. Female 2.8 - 3 mm. Male unknown.

**Coloration.** Head and mesosoma black, antenna yellowish, except scape, brown, metasoma pale brown. Legs pale yellow, except coxae, brown.

**Antenna.** (Fig. 1E) *Female.* Strongly clavate, 11 flagellomeres, antennal formula: 8(4): 4(4): 5(3): 3(3): 3(3): 3(3.5): 4(5): 5(6): 5(6): 6(5): 5(6): 5(6): 7(4). Placoid sensillae from F7 to terminal segment.

**Mesosoma.** Mesoscutum slightly striate. Notauli complete of uniform width. Antero-admedian lines weak. Median mesoscutal line very short. Scutellar foveae round to subquadrate, not delimited posteriorly. Mesopleural furrow absent.

**Forewing.** Radial cell 2.4 times longer than wide.

**Metasoma.** Base of T3 with an almost complete hairy ring.

#### Type material.

HOLOTYPE ♀ (ANIC) with the following label data: “AUSTRALIA: Vict. Mt. Donna Buang, 1200m 11–17.i. 80, Eucalyptus-Nothofagus forest, A. Newton, M. Thayer” (white label), “flight intercept window/trough trap” (white label), “AUST. NAT. INS. COLL.” (green label), “Holotype *Mikeius clavatus* P-V & R-O” (red label). PARATYPE ♀ (ANIC) with the following labels: “W side Cobungra Hill 20km WbyN, Omeo Vic. 27 Feb. 1980, I.D. Naumann J. C. Cardale” (white label), “ex alcohol collection” (white label), “AUST. NAT. INS. COLL.” (green label), “Paratype *Mikeius clavatus* P-V & R-O” (red label).

#### Biology.

Unknown.

#### Distribution.

Victoria, Australia.

#### Etymology.

The specific name refers to the strongly clavate antenna.

### Thrasorinae

Kovalev, 1994

http://species-id.net/wiki/Thrasorinae

Figs 2 3 4 9

#### Type genus:

*Thrasorus* Weld, 1944.

#### Diagnosis.

Distinguished from other figitids by the presence of a circumtorular impression (Figs 2A, D, 3A, 4A, 9A, B) (Table 2); further distinguished from Euceroptrinae by the absence of an areolet in the forewing and the absence of a lateral pronotal carina. Additional characters that distinguish Thrasorinae from other Figitidae can be found in the key to subfamilies below.

#### Comments.

In the redescription of *Thrasorus*, Buffington (2008) erroneously described species of the genus as having 12 flagellomeres in the female antenna; the correct number is 11.

#### Biology.

Unknown.

#### Distribution.

Australia, South America and North America.

#### Included genera:

*Cicatrix*, gen. n.; *Myrtopsen* Rübsaamen, 1908; *Palmiriella*, gen. n., *Scutimica* Ros-Farré, 2007; *Thrasorus* Weld, 1944.

### Cicatrix

Paretas-Martínez gen. n.

urn:lsid:zoobank.org:act:F831C129-F846-4A87-A668-524A2EA64E19

http://species-id.net/wiki/Cicatrix

Fig. 2

#### Type species:

*Cicatrix pilosiscutum* (Girault), **comb. n.**

#### Included species:

*Cicatrix neumannoides*, sp. n., *Cicatrix pilosiscutum* (Girault), *Cicatrix shauffi* (Buffington), comb. n.

#### Diagnosis.

(Table 2) *Cicatrix*, gen. n., is distinguished from *Myrtopsen*, *Palmiriella*, gen. n., and *Scutimica* by having T3 and T4 as separate sclerites (Fig. 2C); in these latter three genera, T3 and T4 are fused into a syntergum (Fig. 3F, 9C). *Cicatrix* is distinguished from *Thrasorus* having horizontally striate microsculpture on the mesoscutum (Fig. 2B, E); *Thrasorus* has a smooth mesoscutum (Fig. 4B).

**Table 2.** Diagnostic table for Mikeius (Mikeiinae, n. subf.) and genera of Thrasorinae.

	Mikeiinae	Thrasorinae				
	*Mikeius*	*Thrasorus*	*Cicatrix*	*Palmiriella*	*Scutimica*	*Myrtopsen*
Circumtorular impression	absent	present	present	present	present	present
Lateral margins of posterior part of pronotal plate	not reaching scutum, not forming an upraised plate	reaching scutum, forming an upraised plate	reaching scutum, forming an upraised plate	reaching scutum, forming an upraised plate	reaching scutum, forming an upraised plate	reaching scutum, forming an upraised plate
Mesoscutum sculpturing	microsculpture horizontally striate	absent, smooth	microsculpture horizontally striate	microsculpture horizontally striate	smooth or with parapsides	microsculpture horizontally striate
T3-T4	T4 2x length of T3	T4 2x length of T3	T4 2x length of T3	fused, syntergum not covering the entire metasoma	fused, syntergum covering the entire metasoma	fused, syntergum covering the entire metasoma
Face sculpturing	absent	carinae on lower face	carinae on lower face	absent	irregularly wrinkled/carinate	irregularly wrinkled/carinate
Posterior margin of scutellum	rounded	rounded	rounded	rounded	emarginate	truncate/emarginate
Pronotum sculpturing	absent	absent	absent	absent	carinate	carinate/<br/> microsculpture
Notauli	complete	complete	complete	complete	incomplete, each forming a large cell	complete

#### Description.

**Length.** Female2.5 – 4.5 mm.Male unknown.

**Coloration.** The entire body with the same coloration, light brown or chestnut depending on the specimen.

**Head** (Fig. 2A, D). Face and frons with abundant setae . Face with transverse carinae, strong across entire face, or only marked at lateral sides of face, smoother, tending towards strigae. Clypeus distinctly projected anteriorly, curved ventrally, clypeopleurostomal lines well developed. Malar furrow coriaceous. Occiput and genae smooth without carinae. Circumtorular impression present.

**Antennae** (Fig. 2F). *Female.*Filiform, with 10 or 11 flagellomeres.

**Mesosoma** (Fig. 2B and E). Pronotal carinae reaching anterior margin of mesoscutum, forming small plate, conspicuous but not projected, concave dorsomedially. Mesoscutum horizontally striate. Notauli complete, of uniform width to slightly wider posteriorly. Parascutal sulcus wide only in basal half. Lateral basal impressions conspicuous. Antero-admedian lines weak. Median mesoscutal line absent, short or long. Scutellum rugose; scutellar foveae round, subtriangular or subquadrate; interfoveal carina absent. Mesopleural furrow conspicuous. Propodeal carinae wide, curved. Pronotum, mesoscutum, scutellum, mesopleural triangle and metapleura all covered with sparse/dense setae.

**Forewing.** Short setae present on wing surface and along margins. Radial cell closed along anterior margin, two times longer than wide, R2 almost straight; areolet absent.

**Legs**. Metatibia with two spurs, sub-equal in length, not exceeding one-half length of tarsomere 1.

**Metasoma** (Fig. 2C). Petiole short. Base of T3 with patches of setae or an almost complete hairy ring. Tergite 3 smaller than T4; T4 four large, covering almost entire metasomal surface; remaining terga short, telescoped within T4; entire metasoma shiny, smooth. Hypopygium and ventral spine visible.

#### Biology.

Unknown.

#### Distribution.

Australia.

#### Etymology.

From the Latin word *cicatrix*, meaning “scar”, refering to the carinae that resemble a scar through the face. Gender is masculine.

#### Taxonomic comments.

Girault (1929) described *Amblynotus* *pilosiscutum*, and Weld (1952) transferred the species to *Melanips*. This species has the circumtorular impression and thus belongs to Thrasorinae. However, the results of the phylogenetic analysis and the diagnostic characters summarized above indicate that this species cannot be accommodated by any currently recognized genus, thus we describe *Cicatrix*, gen. n., to contain *Cicatrix pilosiscutum* (Girault) as well as *Cicatrix neumannoides*, sp. n., and *Cicatrix schauffi* (Buffington), comb. n.

### Cicatrix pilosiscutum

(Girault) comb. n.

http://species-id.net/wiki/Cicatrix_pilosiscutum

Fig. 2A

Amblynotus pilosiscutum Girault, 1929
Melanips pilosiscutum (Girault) Weld, 1952

#### Diagnosis.

Differs from *Cicatrix neumannoides* and *Cicatrix schauffi* by having female antenna with 11 flagellomeres (these two species having female antenna with 10 flagellomeres, (Fig. 2F, *Cicatrix schauffi*)), much stronger carinae crossing the entire face (Fig. 2A) (only marked at lateral sides of the face in the other two species, and being smoother, more like strigae), and by lacking a median mesoscutal impression (present and long in *Cicatrix schauffi* comb. n. (Fig. 2B), short in *Cicatrix neumannoides* sp. n. (Fig. 2E)).

#### Redescription.

As in generic description (see above) with the following specific characters: **Length.** Female 4.4 mm. Male unknown.

**Coloration.** Completely light brown except mesosoma, which is dorsally dark.

**Head.** (Fig. 2A) Frons and face with piliferous punctures; strong transverse carinae crossing the entire face.

**Antenna.** *Female*. 11 flagellomeres, antennal formula: 9(4): 5(3): 10(3): 9(2.5): 8.5(2.5): 8(3): 8(3): 8(3): 7(3): 5(3): 5(3): 4.5(3): 7.5(3). Placoid sensillae absent on basal half of F1 to F4, scarce on dorsal half; abundant from F5 to F11.

**Mesosoma.** Median mesoscutal impression absent. Scutellar foveae subtriangular.

#### Type material.

HOLOTYPE ♀ (QM) with the following labels: “25. 10. 23, National Pk., Q. H. Hacker.” (white label), “HOLOTYPE” (pink label), “*Amblynotus pilosiscutum* ♀, Type Girault” (white label, handwritten), “*Xyalophoroides pilosiscutum* (Gir), E. F. Riek det 1953” (white label, handwritten), QM Reg. No. T99348” (yellow label), “*Cicatrix pilosiscutum* P-M det-2009” (white label).

#### Biology.

Unknown.

#### Distribution.

Australia. Label data suggest the single specimen was taken in Royal National Park in Sydney.

### Cicatrix schauffi

(Buffington) comb. n.

http://species-id.net/wiki/Cicatrix_schauffi

Fig. 2B–D and F

Mikeius schauffi Buffington, 2008.

#### Diagnosis.

Similar to *Cicatrix neumannoides*, sp. n., in having female antenna with 10 flagellomeres (Fig. 2F) and a face horizontally striate only on the lateral areas (Fig. 2D) (*Cicatrix pilosiscutum*, comb. n., has female antenna with 11 flagellomeres and much stronger carinae crossing the entire face), but differs from *Cicatrix neumannoides* sp. n. by having a long median mesoscutal impression and subtriangular scutellar foveae (Fig. 2B).

#### Redescription.

As in generic description (see above) with the following specific characters: **Length.** Female: 3.9 mm. Male unknown.

**Coloration.** Completely light brown.

**Head.** (Fig. 2D) Frons with piliferous punctures, face horizontally striate only on lateral areas.

**Antenna.** (Fig. 2F) *Female*. 10 flagellomeres, antennal formula: 6(2): 4(3): 5(3): 4(3): 4(3): 4.2(3.1): 4.2(3.1): 4.3(3.3): 5.2(3.3): 4.6(3.3): 3.5(3.3): 6(4). Placoid sensillae present from F4, abundant from F6 through terminal segment.

**Mesosoma.** (Fig. 2B) Median mesoscutal impression long, one-third length of scutum. Scutellar foveae irregular, subquadrate and not delimited posteriorly.

**Type material.** HOLOTYPE ♀ (ANIC) with the following label data: “23.36S 133.35E 32 km WNW of Alice Springs, NT 8 Oct. 1978 J:C: Cardale” (white label), “ex alcohol collection” (white label), “AUST. NAT. INS. COLL.” (green label). “HOLOTYPE, *Mikeius* *schauffi*, Buffington” (red label), “*Cicatrix schauffi* P-M det-2009” (white label).

#### Biology.

Unknown

#### Distribution.

Central Australia.

#### Taxonomic comments.

The circumtorular impression present in this species indicates that it belongs in Thrasorinae, not in Mikeiinae. We transfer this species to *Cicatrix* gen. n., because it possesses all the diagnostic characters of that genus.

### Cicatrix neumannoides

Paretas-Martínez & Restrepo-Ortiz sp. n.

urn:lsid:zoobank.org:act:1A6D286B-94AF-43F8-945B-D9AF124EEC57

http://species-id.net/wiki/Cicatrix_neumannoides

Fig. 2E

#### Diagnosis.

Similar to *Cicatrix schauffi*, comb. n., having female antenna with 10 flagellomeres and a face with horizontal strigae only on the lateral areas (*Cicatrix pilosiscutum* comb. n. has female antenna with 11 flagellomeres and much stronger carinae crossing the entire face), but differs from *Cicatrix schauffi* comb. n. by having short median mesoscutal impression and rounded scutellar foveae (Fig. 2E).

#### Description.

As in generic description (see above) with the following specific characters.

**Length.** Female: 2.9 to 3.0 mm. Male unknown.

**Coloration.** Shiny chestnut, scutum darker in center.

**Head.** Frons and face with piliferous punctures; face with a few carinae from internal margin of eye reaching center of face.

**Antenna**. *Female*. 10 flagellomeres, antennal formula: 6(2): 4(2.8): 6(2.5): 4.1(2.8): 4.1(2.8): 4(3): 4(3): 4(3): 4.8(3.1): 3.8(3.3): 3.5(3.3): 5.6(4). Placoid sensillae starting from F4, F4 to F6 are scarce, abundant from F7-F10.

**Mesosoma**. (Fig. 2E) Median mesoscutal impression short, only indicated basally, not reaching one-fifth length of scutum. Scutellar foveae rounded.

#### Etymology.

The specific name *neumannoides* means “related to neumanni”, referring to the fact that the specimens used to describe this species were previously included in the type series of *Mikeius neumanni*.

#### Type material.

HOLOTYPE ♀ (ANIC) with the following labels: “AUSTRALIA: NSW Peak Hill Range, Braidwood, Cooma Road, At top of pass. 30 December 1994. A. Sundholm & R de keyzer. On *Acacia* *dealbata*” (white label), “AUST. NAT. INS. COLL.” (green label), “*Mikeius neumanni* Det. M. L. Buffington 2008” (white label), “Holotype *Cicatrix neumannoides* P-M & R-O” (red label)”. PARATYPE ♀ (ANIC) with the following labels: “Crowea St. For. nr Pemberton W.A. Nov.-Dec. 1978 S.J. Curry Malaise trap open forest” (white label), “AUST. NAT. INS. COLL.” (green label), “Paratype *Cicatrix neumannoides* P-M & R-O” (red label)”.

#### Biology.

Unknown; label data suggests an association with *Acacia*.

#### Distribution.

New South Wales and Western Australia, Australia.

#### Taxonomic comments.

Although Buffington (2008) recognized two specimens of *Mikeius neumanni* in the collection at ANIC, he used only one specimen in his description of the taxon, designating it as the holotype. The species *neumanni* (based on the holotype) is transferred to *Palmiriella*, gen. n., below, and the second specimen, in addition to another specimen discovered in ANIC, belongs to *Cicatrix*.

### Palmiriella

Pujade-Villar & Paretas-Martínez gen. n.

urn:lsid:zoobank.org:act:5F540007-4494-49BF-A619-F0A54F5CE41E

http://species-id.net/wiki/Palmiriella

Fig. 3

#### Type species:

*Palmiriella neumanni* (Buffington), comb. n., by present designation and monotypy.

#### Diagnosis.

(Table 2) *Palmiriella*, gen. n., can be distinguished from other thrasorines by having the face smooth, without any sculpturing (Fig. 3A); in *Scutimica* and *Myrtopsen*, the face is irregularly sculptured (Fig. 9A, B); in *Cicatrix* and *Thrasorus*, strong transverse carinae are present crossing the entire face or on lateral areas (Fig. 3A, D, 4A). *Palmiriella* is further differentiated from other thrasorines by having metasomal T3 and T4 fused into a syntergum, but not covering the entire metasomal surface (Fig. 3F); in *Scutimica* and *Myrtopsen*, a syntergum covering the entire metasomal surface is present (Fig. 9C); in *Cicatrix* and *Thrasorus*, T3 and T4 are separate sclerites (syntergum absent) (Fig. 2C, 4F). Additionally, *Palmiriella* is distinguished from *Scutimica* and *Myrtopsen* by having the scutellum posteriorly rounded (*Scutimica* and *Myrtopsen* have an emarginate/truncate scutellum, Fig. 9D, E), and pronotum not sculptured nor projected (strongly carinate and projected in *Scutimica* (Fig. 9F), with microsculpture or carinate in *Myrtopsen* (Fig. 9G)); from *Thrasorus* by having horizontally striate microsculpture on the mesoscutum (Fig. 3B) (mesoscutum smooth in *Thrasorus*, Fig. 4B).

#### Description.

See description, biology and distribution of type species below.

#### Etymology.

The new genus is dedicated to our colleague and good friend Palmira Ros-Farré, who has helped us for many years with our little wasps. Gender is feminine.

#### Taxonomic comments.

**Figure 5.**
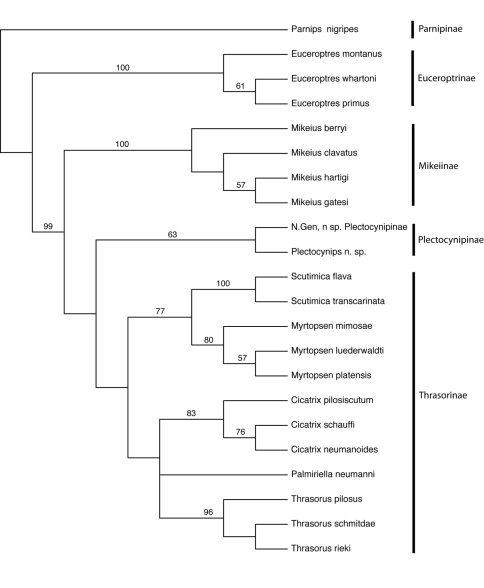
Cladogram of Euceroptrinae, Mikeiinae, Plectocynipinae, and Thrasorinae. Numbers above branches indicate bootstap support. CI=0.58; RI=0.73; RC=0.43. Strict consensus of 2 trees, L=190.

The holotype of *Mikeius neumanni* Buffington, unlike the other species included in *Mikeius*, does have the circumtorular impression diagnostic for Thrasorinae. For this reason, this species is transferred from *Mikeius* to the new thrasorine genus *Palmiriella*. Characters summarized in the diagnosis below and phylogeny in Fig. 5 justify the erection of the new genus.

### Palmiriella neumanni

(Buffington) comb. n.

http://species-id.net/wiki/Palmiriella_neumanni

Fig. 3A–F

Mikeius neumanni Buffington, 2008.

#### Description.

**Length.** Female 3.2 mm. Male unknown.

**Coloration.** Head and mesosoma black, antennae yellowish except scape, brown, metasoma medium brown. Legs light yellow except tibia and metatarsi, brown.

**Head** (Fig. 3A, F). Frons and face with piliferous punctures and abundant setae. No transverse carinae or strigae on face. Clypeus distinctly projected anteriorly, curved ventrally, clypeopleurostomal lines well developed. Malar space with conspicuous, coriaceous, striate band. Vertex in dorsal view with small piliferous punctures. Occiput and genae smooth without carinae. Circumtorular impression present.

**Antenna** (Fig. 3D). *Female*. 11 flagellomeres; antennal formula: 7(4): 4(4): 4(3): 4.5(3): 4.5(3): 4.5(3): 4(3): 4(3): 4(3): 3(3): 3(3): 3(3): 5(4). Placoid sensillae from F7 to terminal segment.

**Mesosoma** (Fig. 3B, C, E). Pronotal carinae reaching scutum, forming small plate, conspicuous but not projected, concave dorsomedially. Mesoscutum horizontally striate. Notauli complete of uniform width. Parascutal sulcus wide only in basal half. Lateral basal impression conspicuous. Antero-admedian lines weak, reaching anterior one-third of mesoscutum. Median mesoscutal impression short and weak. Scutellum rugose; scutellar foveae triangular; interfoveal carina absent. Mesopleural furrow present. Propodeal carinae present. Pronotum, mesoscutum, scutellum, mesopleural triangle and metapleura all covered with sparse/dense setae.

**Forewing.** Short setae present on wing surface and along margins. Radial cell closed, 2.3 times longer than wide; R2 almost straight, basal vein distally widening; areolet absent.

**Legs**. Metatibia with two spurs, sub-equal in length, not exceeding one-half length of tarsomere 1.

**Metasoma** (Fig. 3F). Petiole very short, almost not visible. T3 and T4 fused into a syntergum, not covering the entire metasomal surface; remaining terga short, telescoped within T4; entire metasoma shiny and smooth. Hypopygium and ventral spine visible. Base of syntergum with only some scattered setae.

#### Type material.

HOLOTYPE ♀ (ANIC) with the following labels: “Mt Nebo, S. E. Qld, 24. Xi. 1970, S. R. Monteith” (white label), “AUST. NAT. INS. COLL.” (green label). “HOLOTYPE, *Mikeius* *neumanni*, Buffington” (red label), “*Palmiriella neumanni* P-V & P-M det-2009” (white label).

#### Biology.

Unknown.

#### Distribution.

Queensland, Australia.

### Thrasorus

Weld, 1944

Fig. 4

#### Type species:

*Thrasorus pilosus* Weld, 1944.

#### Included species:

*Thrasorus pilosus* Weld, *Thrasorus rieki*, sp. n., *Thrasorus schmitdae* Buffington.

### Thrasorus rieki

Paretas-Martínez & Pujade-Villar sp. n.

urn:lsid:zoobank.org:act:BCD3677F-EA0D-4D37-B62B-F093CEDF7B02

http://species-id.net/wiki/Thrasorus_rieki

Fig. 4B

#### Diagnosis.

Differs from other species of *Thrasorus* by having small scutellar foveae not clearly defined in posterior margin (Fig. 4B); other species of *Thrasorus* have scutellar foveae clearly delimited in the entire circumference (Fig. 4D). Further differs from other *Thrasorus* species by having a well-defined median mesoscutal impression (arrow, Fig. 4B); in other *Thrasorus*, the impression is not present, or at most, a very small incision can be seen (Fig. 4D).

#### Description.

**Length.** Female: 3.0–3.2 mm; males: 3.2–3.3 mm.

**Coloration.** Head and mesosoma black, antennae brown, and metasoma pale brown. Legs pale yellow except coxae, brown.

**Head.** (Fig. 4A) Frons and face with abundant setae and piliferous punctures; space between clypeus and compound eye with carinae. Malar furrow conspicuous, coriaceous and striate. Occiput smooth; genae with strong striae. Vertex in dorsal view with small piliferous punctures. Circumtorular impression present.

**Antenna.** *Female.* (Fig. 4G) 11 flagellomeres, antennal formula: 6(3): 2(2): 5(2): 4(2): 4(3): 4(3): 4(3): 4(3): 4(3): 3(3): 3(3): 3(3): 5(4). Placoid sensillae from F4 to terminal segment. *Male.* (Fig. 4C) 12 flagellomeres, antennal formula: 7(3): 3(2): 5(2): 4(3): 4(3): 4(3): 4(3): 4(3): 4(3): 4(3): 4(3): 4(3): 4(3): 5(3). Placoid sensillae starting from F1.

**Mesosoma** (Fig. 4B, E). Lateral margins of pronotal plate reaching the scutum, forming a small plate conspicuous but not projected, concave dorsomedially, with piliferous punctures. Mesoscutum smooth and shiny, with piliferous punctures. Notauli complete, very narrow anteriorly and much wider posteriorly. Parascutal sulcus wide only in basal half. Lateral basal impressions weak. Antero-admedian lines very weak. Median mesoscutal impression well defined but not clearly delimited anteriorly. Scutellum smooth on anterior falf and centre, rugose posteriorly; scutellar foveae small subtriangular, not clearly delimited posteriorly; interfoveal carina absent. Mesopleural furrow present but not conspicuous. Propodeal carinae present. Pronotum, mesoscutum, scutellum, mesopleural triangle and metapleura not very pubescent, only some sparse setae.

**Forewing.** Short setae present on wing surface and along margins. Radial cell closed, 1.9 times longer than wide; R2 almost straight; areolet absent.

**Legs**. Metatibia with two spurs, sub-equal in length, not exceeding one-half length of tarsomere 1.

**Metasoma**. (Fig. 4F) Petiole short. Base of T3 with an almost complete hairy ring. Tergite 3 smaller than T4; T4 four large, covering almost entire metasomal surface; remaining terga short, telescoped within T4; entire metasoma shiny and smooth.

#### Material examined.

HOLOTYPE ♀ (ANIC; marked by a red spot, on a pinned card with six other specimens of the same taxon) with the following labels: “Out of large galls on mullee acacia On 18–1-16” (handwritten below the label with the insects), “Thrasorus berlesei (Grlt) Riek det” (white label, handwritten), “sp 7 (berlesei) det ML Buffington 2006” (white label), “Holotype *Thrasorus rieki* P-M & P-V det-2009” (red label). PARATYPES: 4 ♂ and 1 ♀ (on the same pinned card as the holotype) with the same data as the holotype, “Paratype *Thrasorus rieki* P-M & P-V det-2009” (red label); 1 ♂ and 5 ♀ (ANIC) (on a pinned card together with 6 Chalcidoidea specimens) with the following labels: “Out of Acacia galls ???? 19.1.16 QLD” (handwritten below the label with the insects), “AUST. NAT. INS. COLL.” (green label), “Paratype *Thrasorus rieki* P-M & P-V det-2009” (red label); 1 ♀ (QM) with the following labels: “Amblynotus berlesei ♀ Girault types” (white label handwritten), “HOLOTYPE” (pink label), “Thrasorus berlesei (Gir) EF Riek det 1953” (white label handwritten), “QM reg. No. T99347” (yellow label), “Paratype *Thrasorus rieki* P-M & P-V det-2009” (red label).

#### Biology.

Unknown host on *Acacia* galls (based on label data).

#### Distribution.

Australia, Queensland.

#### Etymology.

Named after E.F. Riek, who worked before us on Australian Cynipoidea.

#### Taxonomic comments.

In the QM, there is one specimen labelled as ‘*Amblynotus berlesei*’ by Girault. In ANIC, there are six specimens on one large card with a determination label placed by Riek, stating that taxon is ‘*Thrasorus berlesei* (Grlt)’. But as Buffington (2008) pointed out, this species was never published by Girault nor Riek. As this name is a *nomen nudum* after Buffington (2008), we described it as a new species. In ANIC, there is another large card that has six specimens of *Thrasorus rieki*, sp. n., mixed with Chalcidoidea specimens.

## Discussion

Ros-Farré and Pujade-Villar (2007) described the single synapomorphy that supports the monophyly of Thrasorinae: the circumtorular impression (Figs 2A, D, 3A, 4A, 9A, B), a clear and marked impression around the upper half of each torulus. Though the shape of this impression is variable among genera and species of Thrasorinae, the presence of this character is constant in all the species of the subfamily and thus must be considered as a strong synapomorphy of the Thrasorinae. The circumtorular impression in *Scutimica* is more laterally directed and wide, with a few ‘ribs’ inside (Fig. 9A); in *Myrtopsen* the impression can vary from very tight and deep in some species (Fig. 9B) to a state similar to that in *Scutimica*; in *Palmiriella* (Fig. 3A) and *Thrasorus* (Fig. 4A), the impression is well defined, deep, wide, and delimited by a small crest; in *Cicatrix*, the impression is also wide but not delimited by a crest, and it is deeper in some species (Fig. 2A) than in other (Fig. 2D). Three genera previously included in Thrasorinae that do not possess this character have recently been moved to new subfamilies: *Plectocynips* and *Pegascynips* to the Plectocynipinae (Ros-Farré and Pujade-Villar 2007), and *Euceroptres* to the Euceroptrinae (Buffington and Liljeblad 2008).

*Mikeius*, described by Buffington (2008), was included in the Thrasorinae based on its general morphology and its association with chalcidoid galls. However, as shown here, *Mikeius* does not have the circumtorular impression (Fig. 1A), diagnostic of Thrasorinae. Further, *Mikeius* possesses a character not present in the other subfamilies treated here: *projected* *pronotal plate lacking*, the area instead being marked by two carinae in the median part of the pronotum that do not reach the anterior margin of the mesoscutum (Fig. 1G). A similar state can be found in *Euceroptres* (Buffington and Liljeblad 2008) and *Lonchidia* (Fig. 8E). In both *Mikeius* and *Euceroptres*, the submedial pronotal depressions of the plate (lateral fovea of pronotum, Buffington 2009) are present and are open laterally. Overall, the impression of the observer is that the pronotal plate is lacking entirely; we argue here that the plate is present, evidenced by the presence of the submedial pronotal depressions, as well as the anterior part of the pronotal plate (portion of plate ventral to submedial pronotal depressions). The portion of the plate that is reduced is the posterior part of the pronotal plate, or the portion of the plate dorsal to the submedial pronotal depressions. The arrow in Fig. 1F shows where the lateral portion of the dorsal part of the pronotal plate fades into the remaining cuticle, just ventrad of the anterior margin of the mesoscutum. Further, the dorsal margin of the plate is completely undefined, as compared with the state found in *Palmiriella* (Fig. 3E) and *Thrasorus* (Fig. 4B). Unfortunately, to fully appreciate this character, the head must be removed from a specimen in hand.

The morphology of the metasoma is a very important character and is frequently used in all Figitidae subfamilies to separate different genera. Within Thrasorinae, there are two main metasomal morphologies: T3-T4 free (*Thrasorus*, *Cicatrix*), and T3-T4 fused into a syntergum (*Palmiriella*, *Scutimica*, *Myrtopsen*). The primary difference between *Thrasorus* and *Cicatrix* is the sculpturing of the mesoscutum. Though the sculpturing on the mesoscutum can be variable in other groups of Figitidae, in the ‘pool’ of genera treated in this paper, mesoscutal sculpture is useful and unique character. *Thrasorus* is the only genus, not only among Thrasorinae but also among all the genera previously included in this subfamily (*Plectocynips*, *Pegascynips*, *Euceroptres*, *Mikeius*), that aside from notauli, lacks sculpturing of any kind (microsculpture, carinae or parapsides) in the mesoscutum; we believe that this character is enough to justify the separation of *Thrasorus* from *Cicatrix* and the other thrasorines.

**Figure 6.**
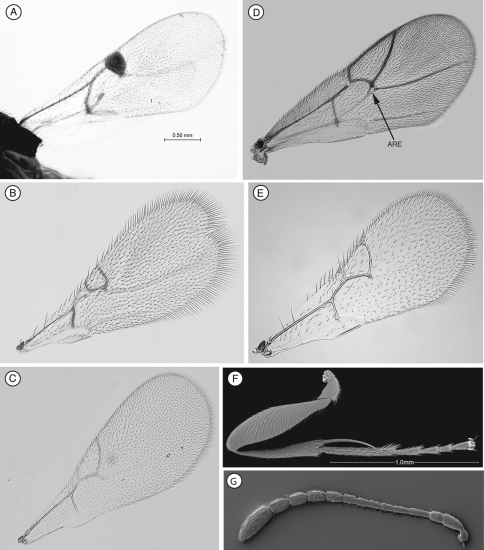
Characteristics of Figitidae: forewing, leg and antenna **A** *Pycnostigmus rostratus* (Pycnostigminae) **B** *Emargo* sp. (Emargininae) **C** *Phaenoglyphis* sp. (Charipinae) **D** *Euceroptres montanus* (Euceroptrinae) **E** *Agrostrocynips diastrophus* (Eucoilinae) **F** hindleg, *Plectocynips pilosus* (Plectocynipinae) **G** female antenna, *Lonchidia* sp. (Figitinae).

**Figure 7.**
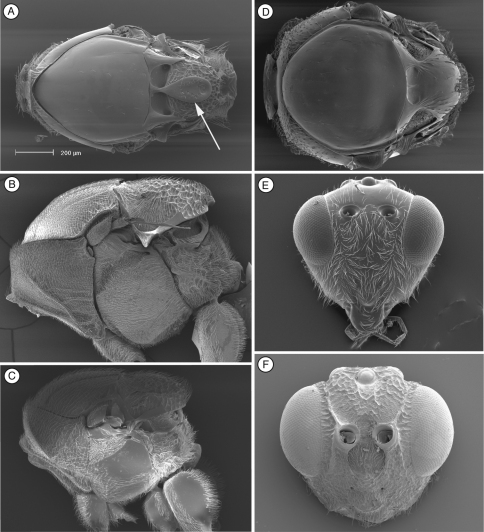
Characteristics of Figitidae: mesosoma and head **A** *Trybliographa rapae* (Eucoilinae) **B** *Parnips nigripes* (Parnipinae) **C** *Euceroptres montanus* (Euceroptrinae) **D** *Phaenoglyphis* sp. (Charipinae) **E** *Anacharis* sp. (Anacharitinae) **F** *Aspicera* sp. (Aspicerinae).

**Figure 8.**
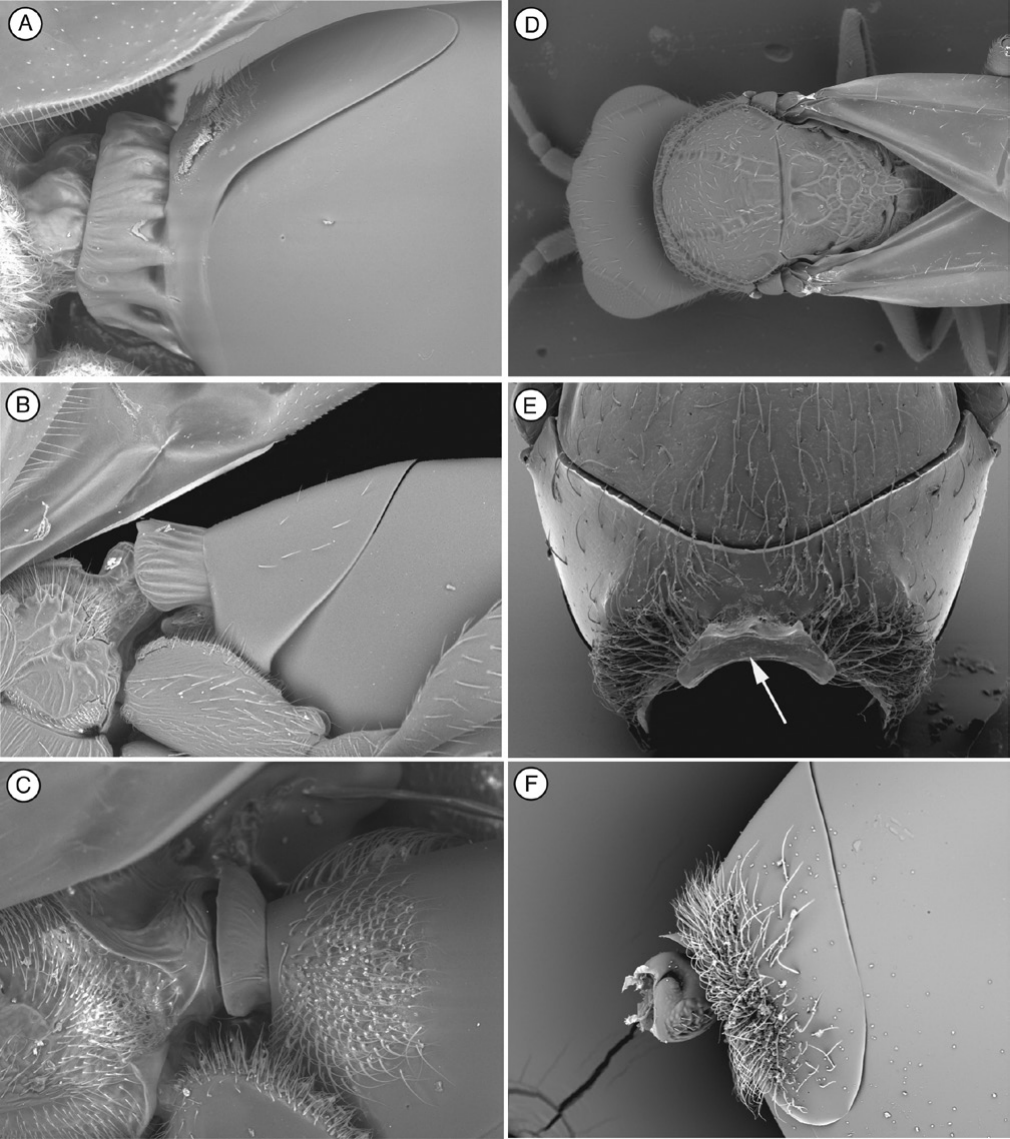
Characteristics of Figitidae: mesosoma and metasoma **A** metasomal tergum 2 and 3, *Callaspidia* sp. (Aspicerinae) **B** metasomal tergum 2 and 3, *Figites* sp. (Figitinae) **C** metasomal tergum 2 and 3, *Melanips opacus* (Figitinae) **D** head and mesosoma, *Xyalaspis* sp. (Anacharitinae) **E** pronotum, antero-dorsal view, *Lonchidia* sp. (Figitinae), arrow indicating anterior half of pronotal plate **F** metasomal tergum 2 and 3 *Mikeius hartigi* (Mikeiinae).

**Figure 9.**
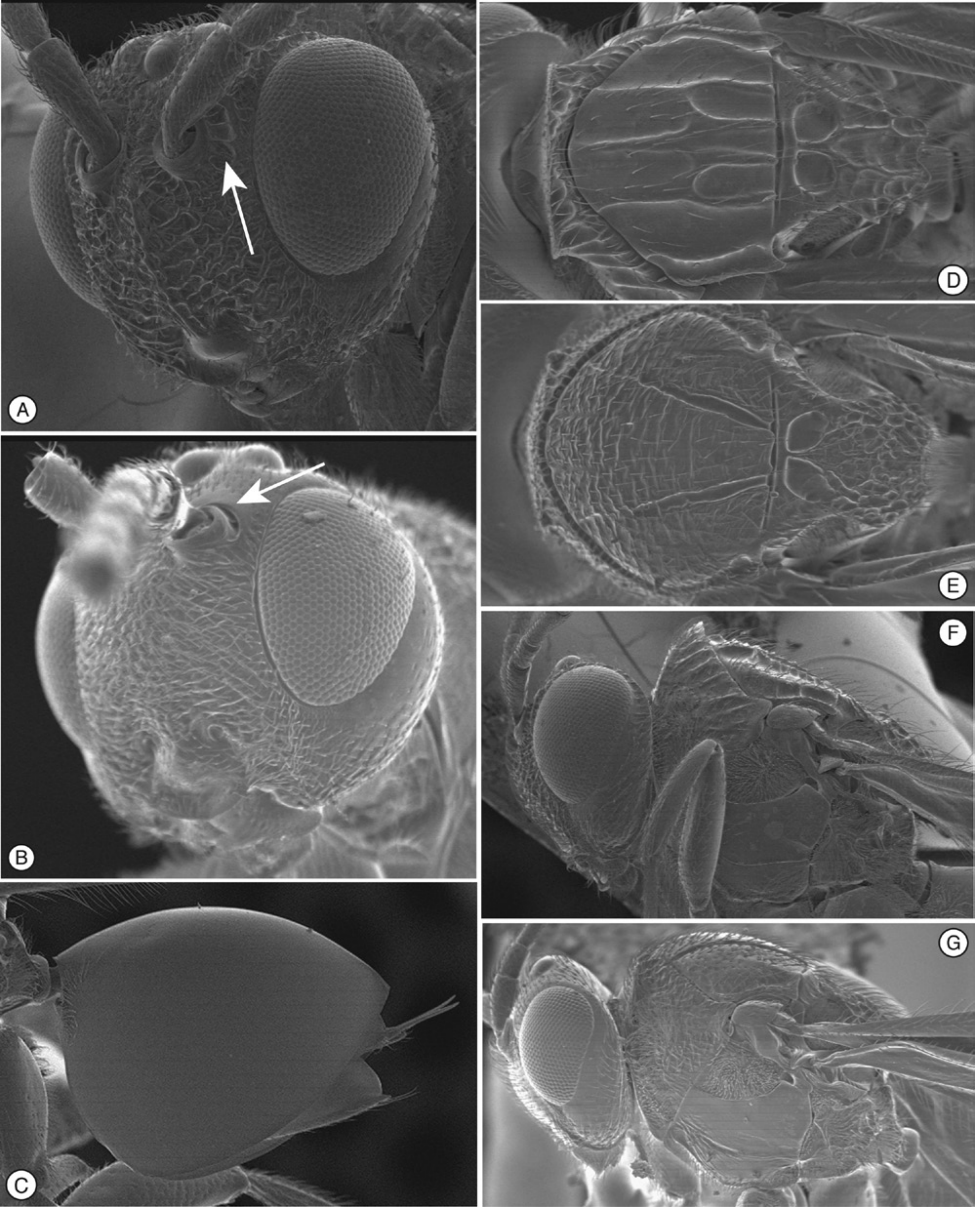
Characters of *Scutimica* and *Myrtopsen* **A** head, *Scutimica transcarinata* **B** head, *Myrtopsen luedervaldti* **C** metasoma, *Myrtopsen* sp. **D** mesosoma in dorsal view, *Scutimica flava* **E** mesosoma in dorsal view, *Mikeius mimosae* **F** mesosoma in lateral view, *Scutimica transcarinata* **G** mesosoma in lateral view, *Mikeius punctuatus*.

The characters that differentiate *Palmiriella* from *Scutimica* and *Myrtopsen* are detailed in the diagnosis of the genus (see above). The combination of the smooth face (*Palmiriella* being the only genus among Thrasorinae and genera previously included in the subfamily lacking any kind of sculpture on face), shape of syntergum T3-T4, shape of scutellum, shape of pronotum, and absence of sculpturing on pronotum, distinguish *Palmiriella* from *Scutimica* and *Myrtopsen*. The differences between *Scutimica* and *Myrtopsen* have already been remarked and discussed in Ros-Farré and Pujade-Villar (2007).

The results of the phylogenetic analysis are summarized in Fig. 5. Two trees of length 190 were recovered, with a CI of 0.58, RI of 0.73, and RC of 0.43. All subfamilies treated here were recovered as monophyletic, with following pattern of relationship: Parnipinae (Euceroptrinae (Mikeiinae (Plectocynipinae (Thrasorinae)))). It is clear that *Mikeius* renders the Thrasorinae paraphyletic, supporting the description of Mikeiinae, and that *Cicatrix* and *Palmiriella* are distinct clades. Erecting a new subfamily for a single genus is not desirable, but the only alternative to this while respecting the clades recovered in the phylogenetic analysis would be grouping together *Mikeius*, *Palmiriella*, *Thrasorus*, *Cicatrix*, *Scutimica*, *Myrtopsen*, *Plectocynips* and *Pegascynips* in a single subfamily; we feel this grouping is undesirable from the standpoint of predictability, since these genera contain species possessing markedly different biological and morphological attributes, and still would lack a single common diagnostic character for all of them. As currently defined, each of the subfamilies recognized here has its own diagnostic character: long metatibial spur for Plectocynipinae, circumtorular impression for Thrasorinae and two carinae in median area of pronotum not forming a projected pronotal plate for Mikeiinae.

The Thrasorinae from Australia are one of the most poorly known groups of figitids. More field data and specimens would help to clarify the status of this group and some taxa described here. However, there is no single researcher in Australia dedicated to the study of Cynipoidea, and workers on Figitidae wanting to study the systematics of this group must rely on ‘rare’ specimens coming from non-target collections while pursuing the sampling of other groups. The study we present here has been done with all the thrasorines and *Mikeius* that have been collected, curated, and deposited in museums worldwide.

## Key to figitid subfamilies of the World

**Table:** 

1	Radial cell secondarily sclerotized forming a pseudostigma (Fig. 6A); Afrotropical and southwestern Palaearctic regions, rarely collected	Pycnostigminae
–	Radial cell (Fig. 6 B–E) not sclerotized, forming a typical wing cell	2
2	Scutellum with an oval, tear-drop shaped, or elongate elevated plate dorsally (arrow, Fig. 7A); scutellar plate equipped with a glandular release pit medially or posteriorly; parasitoids of Diptera: Cyclorrhapha; Cosmopolitan	Eucoilinae
–	Scutellum different or occasionally with raised carinae defining a central area but never with an elevated plate equipped with a glandular release pit dorsally (Figs 7 B–D and 8D)	3
3	Metatibial spur at least half the length of metatarsomere 1 (Fig. 6F); associated with hymenopteran galls in *Nothofagus* forests in the Neotropical Region	Plectocynipinae
–	Metatibial spur at most 1/4 length of metatarsomere 1	4
4	Apex of forewing deeply bilobed (Fig. 6B); Pantropical, rarely collected	Emargininae
–	Apex of forewing rounded (Fig 6 C–D)	5
5	Areolet present on forewing (Fig. 6D); base of metasoma always glabrous	6
–	Areolet absent on forewing (Fig. 6 C, E); base of metasoma setose or glabrous	7
6	Mesopleuron completely strigose, with no indication of a distinct mesopleural furrow (Fig. 7B); parasitoids of *Barbotinia* (Cynipidae) in *Papaver* (Papaveraceae); Palaearctic, Mediterranean Region	Parnipinae
–	Mesopleuron dorsally smooth, ventrally striate along the mesopleural furrow, mesopleural furrow distinct (Fig. 7C); parasitoids of *Andricus* (Cynipidae) in *Quercus* (Fagaceae) in the Nearctic Region	Euceroptrinae
7	Head triangular in anterior view (Fig. 7E), always wider than the mesosoma (in dorsal view; Fig. 8D); mouth region small, with mandibles broadly overlapping (Fig. 7E); parasitoids of Neuroptera; Cosmopolitan	Anacharitinae
–	Head squared or rounded in anterior view (Figs 1A, 2 A & D, 7F), wider, equal to, or narrower than the mesosoma; mouth region broadened, mandibles larger and not overlapping as extensively (Figs 1A & 7F)	8
8	Facial impression present (Fig. 7F); third metasomal tergum distinctly saddle shaped with posterolateral margin concave and central part almost tongue-like (Fig. 8A); parasitoids of Diptera: Syrphidae; Cosmopolitan	Aspicerinae
–	Facial impression absent (Fig. 1A); third abdominal tergum rounded, not saddle-shaped, with the posterolateral margin usually convex, rarely concave (Figs 3F, 4F, 8B–C, F)	9
9	Body lacking transversally carinate sculpture, generally shiny and smooth (Fig. 7D) (*Lytoxysta* is exceptional in havingfine reticulate sculpturing on the head and mesosoma; some species of *Phaenoglyphis* have fine imbricate sculpture on the mesoscutum and scutellum (Paretas-Martínez et al. 2007)); scutellum broadly rounded and without sculpture (Fig. 7D); mesopleural triangle present or absent; notauli absent (Fig. 7D) or present; small insects, typically 1 mm in length; hyperparasites in Aphididae and Psylidae; Cosmopolitan	Charipinae
–	Mesoscutum usually with some transversal macro or microcarinate sculpture (Figs 1F, 2 B & E, 3B, 4D & H, 8D), sometimes smooth or at most piliferous (Fig. 4B); mesopleural triangle always present (Fig. 1B, 3C, 4E); notauli partially to fully present (Fig. 1F, 2B & E, 3B, 4B & D & H, 8D); larger insects, typically greater than 2mm in length	10
10	Circumtorular impression present (CI, Fig. 2A & D, 3A, 4A)	Thrasorinae
-	Circumtorular impression absent (Fig. 1A)	11
11	Second metasomal segment modified into either a collar with strong carinae (Fig. 8A), a carinate sheath (Fig. 8B) or carinate flange Fig. 8C), obscuring part of the petiole in lateral and dorsal view; parasitoids of Diptera: Cyclorrhapha; Cosmopolitan	Figitinae
–	Second metasomal segment small, not heavily sclerotized, typically obscured by the anterior margin of tergite 3 (Fig. 8F); Australian Region, parasitoids of gall inducing Hymenoptera	Mikeiinae, subfam. n.

## Key to genera of Thrasorinae

**Table:** 

1	Metasomal syntergum absent (post-petiolar terga free) (Fig. 2C)	2
–	Metasomal syntergum present (post-petiolar terga fused) (Figs 3F, 9C)	3
2	Mesoscutum with horizontal microsculpture (Fig. 2B, E); face with strong or weak transverse strigae (Fig. 2A, D)	*Cicatrix* gen. n.
–	Mesoscutum smooth, at most with some piliferous punctures (Fig. 4B, D); face without transverse strigae; if present, strigae are weak (Fig. 4A)	*Thrasorus* Weld
3	Metasomal syntergum not covering the entire metasomal surface (Fig. 3F); face without transversal strigae (Fig. 3A)	*Palmiriella*, gen. n.
–	Metasomal syntergum covering the entire metasoma (Fig. 9C); face with strigae (Fig. 9A, B)	4
4	Mesoscutum smooth or with parapsides; notauli incomplete, not reaching pronotum, each one forming a large cell (Figs 4H, 9D). Pronotum sometimes projected, with very strong longitudinal carinae (Figs 4H, 9F)	*Scutimica* Ros-Farré
–	Mesoscutum with microsculpture (only one species with transverse carinae); notauli complete, even if being much larger at the base than close to pronotum (Fig. 9E). Pronotum not projected, striate or with strong irregular carinae (Fig. 9G)	*Myrtopsen* Rübsaamen

## Supplementary Material

XML Treatment for Mikeiinae

XML Treatment for Mikeius clavatus

XML Treatment for Thrasorinae

XML Treatment for Cicatrix

XML Treatment for Cicatrix pilosiscutum

XML Treatment for Cicatrix schauffi

XML Treatment for Cicatrix neumannoides

XML Treatment for Palmiriella

XML Treatment for Palmiriella neumanni

XML Treatment for Thrasorus

XML Treatment for Thrasorus rieki

## Appendix I

List of morphological and biological characters used in analysis

**Note:** The list of morphological and biological characters is available on the *ZooKeys* website as a Microsoft Word file (.doc), doi: 10.3897/zookeys.108.829.app.1).

## Appendix II

Data matrix which includes 79 parsimony-informative characters.

**Note:** The data matrix is available on the *ZooKeys* website as a Microsoft Word file (.doc), doi: 10.3897/zookeys.108.829.app.2).
